# A pediatric cancer patient with suspected chemical coping following high-dose opioid therapy: a case report

**DOI:** 10.1186/s13256-019-2273-7

**Published:** 2019-11-30

**Authors:** Mototsugu Miura, Kenkichi Tsuruga, Yuji Morimoto

**Affiliations:** 1 Department of Anesthesiology and Critical Care Medicine, Hokkaido University Graduate School of Medicine, Hokkaido, Japan; 2 Hokkaido University Hospital Cancer Center, Hokkaido, Japan

**Keywords:** Chemical coping, Opioid, Pediatric, Pseudo-addiction, Anxiety

## Abstract

**Background:**

Chemical coping is an inappropriate method for dealing with stress through the use of opioids; it is considered the stage prior to abuse and dependence. In patients with cancer, it is important to evaluate the risk of chemical coping when using opioids. There are some pediatric opioid use-related tolerances and addictions; however, no mention of chemical coping has been found.

**Case presentation:**

We present a case of an 11-year-old Japanese boy with acute lymphocytic leukemia. After transplantation, he complained of abdominal and articular pain, which are considered as symptoms of graft-versus-host disease; thus, opioid therapy was initiated, and the dose was gradually increased for pain management, resulting in a high dose of 2700 μg/day of fentanyl (4200–4700 μg/day including the rescue dose). After switching from fentanyl to oxycodone injections, he continued to experience pain, and there was no change in the frequency of oxycodone rescue doses. Physically, his pain was considered to have alleviated; thus, there was the possibility of mental anxiety resulting in the lowering of pain threshold and the possibility of chemical coping. Mental anxiety and stress with progress through schooling was believed to have resulted in chemical coping; thus, efforts were made to reduce the boy’s anxiety, and opioid education was provided. However, dose reduction was challenging. Ultimately, with guidance from medical care providers, the opioid dose was reduced, and the patient was successfully weaned off opioids.

**Conclusions:**

When chemical coping is suspected in pediatric patients, after differentiating from pseudo-addiction, it might be necessary to restrict the prescription for appropriate use and to provide opioid education while taking into consideration the emotional background of the patient that led to chemical coping.

## Background

Chemical coping was first proposed by Bruera *et al.* in 1995 as “an inappropriate method of dealing with stress through the use of drugs seen in patients suffering from terminal-phase cancer” [1]. In recent years, chemical coping using opioids to deal with psychological and spiritual distress has been considered the stage prior to abuse and dependence [2]. In patients with cancer who are receiving opioid therapy, a history of alcohol dependence and drug abuse, age < 65 years, psychiatric disturbance, high emotional stress, and limited coping mechanisms are considered as risk factors for chemical coping [2–5]. In a previous report, 18% of adult patients with cancer who were receiving opioids exhibited signs of chemical coping upon evaluation by a palliative care specialist [6].

Simple screening tools, such as the CAGE questionnaire [7] and the Screener and Opioid Assessment for Patients with Pain [8], may be used to assess the risk of chemical coping [3, 9]. However, these assessment tools have been developed on the basis of tools used for alcohol dependency, and whether they can be used in children remains unknown. Appropriate methods of assessment and treatment have not been established yet; in fact, there are some pediatric opioid use-related tolerances and addictions; however, no mention of chemical coping has been found. We report our experience with a pediatric patient with cancer suspected of chemical coping and in whom opioid dose reduction was difficult.

## Case presentation

Our patient was an 11-year-old Japanese boy (height 141 cm, weight 36.5 kg) with acute lymphocytic leukemia. Since the onset of acute lymphocytic leukemia, he had received early-stage intensive chemotherapy, remission therapy, and maintenance therapy; however, because he had a positive test result for minor breakpoint cluster region, umbilical cord blood transplantation was performed. After transplantation, he complained of abdominal and articular pain; his abdominal pain was accompanied by frequent diarrhea. These were considered to represent gastrointestinal symptoms of graft-versus-host disease (GVHD); thus, opioid therapy was initiated. For long-term opioid therapy, few opioid medications provide information on the label regarding the safety and effectiveness of the drug in pediatric patients [10]. We selected fentanyl because chemotherapy for leukemia predisposes the patient to renal dysfunction, and it is considered effective because it has high selectivity for mu 1 receptors in the treatment of mucosal pain [11, 12]. However, the dose of fentanyl was gradually increased for pain management, resulting in dose as high as 2300 μg/day, which required intervention from the palliative care team.

**Fig. 1:**
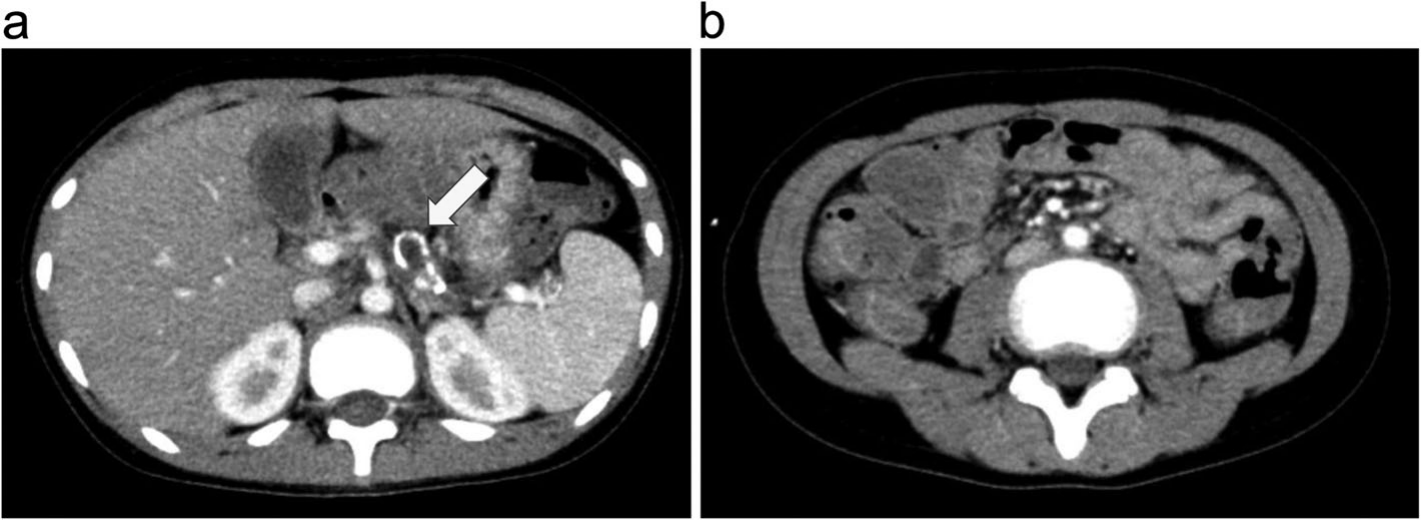
Abdominal imaging findings. **a** Ring-shaped calcification with findings of old fat necrosis in the pancreatic tail (arrow). **b** Mild intestinal edema (graft-versus-host disease findings)

At the time of intervention, the patient was isolated in a sterilized room after transplantation. In addition to the major complaint of pain in the lower left abdomen, upon palpation, the patient complained of pressure pain throughout the abdomen; he also complained of joint pain in the legs when the abdominal pain intensified. Computed tomography revealed changes showing pancreatitis and mild intestinal edema, which was considered to be a sign of GVHD (Fig. 1).

Given the intense acute pain caused by GVHD, the fentanyl dose was increased again to 2700 μg/day; however, the frequency of the rescue dose for pain (equivalent to 1-h dose of continuous infusion) did not decrease below 15 times per day, and continuously increasing the dose did not reduce the frequency of the rescue dose. After the opioid was switched to 90 mg/day of oxycodone injections, the patient continued to experience pain, and there was no change in the frequency of oxycodone rescue doses (Table 1).

**Table 1:** Progress tabel

After umbilical cord blood transplant	Symptom, event	Opioid dose for scheduled use	Number of rescues (continuous infusion at an elevated rate)	Analgesic adjuvant
Day 13	Intervention by the palliative care team	Fentanyl 2300 μg	16	
Day 17	Articular pain in lower limbs in addition to pain in the pain in the left lower abdomen; nausea and fatigue due to GVHD (and opioids?)	Fentanyl2700 μg	19	
Day 19	Diarrhea improved from watery stools to soft stools, but abdominal pain remained unchanged; the opioid was switched to oxycodone for injection.	Oxycodone for injection 90 mg	14	
Day 21	After switching, complaint of pain continued, and the number of rescues remained unchanged.	Oxycodone for injection90 mg	12	
Day 24		Oxycodone for injection100 mg	16	
Day 27	For attending school (in the hospital), syringe pump administration was switched to patient-controlled analgesia.	Oxycodone for injection100 mg	18	
Day 28	The patient showed a tendency toward constipation, and laxatives were adjusted, including naldemedine.	Oxycodone for injection100 mg	15	
Day 44 (early in March)	The patient expressed anxiety about entering junior high school; nausea and fatigue intensified.	Oxycodone for injection84 mg	13	
Day 53	The patient exhibited strong resistance to dose reduction because of fear of possible intensified pain: “No one knows how I am feeling.”	Oxycodone for injection72 mg	14	
Day 64	Multidisciplinary conference	Oxycodone for injection72 mg	13	Duloxetine 10 mg
Day 83	Official entrance ceremony of junior high school (outside the hospital); oral immediate-release oxycodone preparation was prescribed.	Oxycodone for injection72 mg	12	Duloxetine 20 mg
Day 97	Oral immediate-release oxycodone preparation was discontinued; dose reduction was started without telling the dose for scheduled use after consent was obtained from the patient and his mother.	Oxycodone for injection60 mg	12	Duloxetine 20 mg
Day 105	The patient stayed out (his home) overnight on weekends.	Oxycodone for injection54 mg	16	Duloxetine 20 mg
Day 119	The number of rescues did not decrease, but pain did not intensify after reducing the dose for scheduled use.	Oxycodone for injection48 mg	11	Duloxetine 20 mg
Day 121		Oxycodone for injection42 mg	10	Duloxetine 20 mg
Day 134		Oxycodone for injection30 mg	6	Duloxetine 20 mg
Day 136		Oxycodone for injection18 mg	11	Duloxetine 20 mg
Day 137		Oxycodone for injection12 mg	8	Duloxetine 20 mg
Day 139		Oxycodone for injection6 mg	13	Duloxetine 20 mg
Day 143		Oxycodone for injection3 mg	8	Duloxetine 20 mg
Day 148	No complaint of pain; acetaminophen 200 mg and ibuprofen 100 mg were prescribed.	Discontinued		Duloxetine 20 mg
Day 168	Discharged to home			Duloxetine 20 mg
Day 180	No complaint of pain at the outpatient visit; analgesic agents were discontinued, including duloxetine.			Discontinued

*GVHD* Graft-versus-host disease

The patient’s general condition improved, and he did not require isolation. Despite attending school in the hospital, there was no improvement in his complaints of pain, and just before entering junior high school, he expressed anxiety about friends, learning, and whether he would be understood by the teachers. Considering the possibility of opioid overdose in response to complaints of nausea and fatigue, dose reduction was planned; however, he exhibited strong resistance. Furthermore, he became irritable, and his mental instability became evident as exhibited by violent outbursts.

Computed tomography revealed no findings that caused physical pain. His pain was considered to have alleviated; thus, health professionals involved in his care (that is, pediatrician, pediatric psychiatrist, palliative care team, ward nurse, child medical care support provider, and childcare worker) examined the possibility of mental anxiety resulting in the lowering of pain threshold and the possibility of chemical coping.

Expecting to use less opioid, we initiated duloxetine, which exerts an antidepressive effect and adjuvant analgesic effect, at a dose of 10 mg/day. Furthermore, to address the patient’s mental anxiety, a meeting was held with the teacher whose class the patient was expected to attend. The new school staff cooperated so that the patient could attend the same class as his good friends. To address the patient’s drug use, upon suspicion that the sudden increase in blood concentration due to administration of rescue doses of opioid injections could have caused chemical coping, switching to oral opioids was attempted; however, on a pain scale (scale of 0 to 5), the patient assessed that the rescue doses of intravenous oxycodone had reduced his pain from 5 to 1.2 points, whereas the oral oxycodone immediate-release preparation had only reduced pain from 5 to 4.5 points; thus, switching to oral drugs was not successful.

We believed that the rescue dose of intravenous oxycodone resulted in a sudden increase in blood concentration, and the administration of the rescue dose could have been a coping behavior. An explanation regarding opioids in general and the possibility that the number of rescues will not decrease for purposes other than analgesia (such as antianxiety) was shared with the patient and his family members who provided the consent; thereafter, we decided to lower the concentration of intravenous oxycodone without informing the patient of the timing of dose reduction.

After approximately 1 month, the intravenous oxycodone dose was gradually reduced to 3 mg/day; however, there was no major change in the frequency of rescue doses. After the patient was informed that the intravenous oxycodone had been reduced to a dose that had been ineffective as an analgesic, we prescribed 200 mg of acetaminophen and 100 mg of ibuprofen to be taken as needed. Subsequently, oxycodone infusion was discontinued, and the patient did not complain of pain. Thereafter, he expressed no desire for opioid use and was discharged. Currently, he is being treated on an outpatient basis and is opioid-free.

## Discussion and conclusions

Our patient complained of intense pain that was medically difficult to explain, and he requested frequent rescue doses of continuous oxycodone infusion. With the suspicion of chemical coping, we were able to wean the patient from opioids through psychological care and gradually reducing the oxycodone dosage.

When chemical coping is suspected, it is important to differentiate pseudo-addiction, opioid tolerance, and opioid-induced hyperpathia [13]. Pseudo-addiction is defined as the state in which the patient excessively or dramatically complains of pain and frequently seeks analgesics to escape from the inadequately controlled pain [13], which, if misdiagnosed, can lead to insufficient control of pain in a pseudo-dependent patient. In our patient, given the presence of pain associated with GVHD following umbilical cord blood transplantation, pseudo-addiction could explain why there was no change in the complaints of pain despite increasing the opioid dose and the frequency of rescue doses during pain. However, even after mucositis symptoms such as stomatitis and diarrhea improved, the reason that the opioid dose was not reduced might be attributed to chemical coping through the administration of rescue doses of intravenous opioids, which was largely affected by psychological factors such as fear that opioid dose reduction would intensify abdominal pain, loneliness during isolation because of an immunocompromised state, anxiety about entering junior high school, and actual coping behavior (that is, pressing the rescue dose button). However, the possibility of tolerance and opioid-induced hyperalgesia could not be completely ruled out.

To prevent chemical coping, accurately evaluating pain upon opioid introduction, clarifying patient’s history, and verifying whether the frequency of rescue doses has decreased and whether pain is alleviated upon regularly increasing the opioid dose are necessary. When chemical coping is suspected, the health care team may need to be proactive in addressing the patient’s emotional needs, providing proper education on safe opioid use, and monitoring the patient for aberrant behaviors [14].

Our patient was a boy in whom a screening tool was not used at the time of opioid introduction. However, upon suspecting chemical coping, a multidisciplinary conference was held, including a palliative care team, in which the mental status of the patient was shared and a team of medical staff was formed to look after the patient. By reducing the patient’s anxiety through organizing entry into junior high school, we were able to reduce his desire for rescue doses. Education regarding the safe use of opioids was provided by the palliative care specialist. The patient was switched to oral immediate-release oxycodone with the aim of weaning him off continuous drip therapy; however, this attempt failed. Hence, compulsory opioid dose reduction was implemented with the consent of the patient and his family members.

Barglow examined countermeasures for opioid overdose, including improper use, and divided them into three categories (that is, demand reduction [counseling and education about proper opioid use], supply reduction [restricting prescriptions and access so that the patient uses the appropriate dose for pain relief], and harm reduction [medication-assisted treatment {MAT}]) [15]. In our patient, the opioid dosage could not be reduced through demand reduction, but it could be reduced through supply reduction; however, we believe that further examination is warranted to determine whether demand reduction results in poorer treatment outcomes for chemical coping in pediatric patients than in adults. In contrast, MAT for opioid use disorder in adolescents has been reported [16]; thus, dose reduction through MAT using drugs such as buprenorphine might be a good treatment option for chemical coping in children.

## Data Availability

Not applicable.

## Abbreviations

GVHD: Graft-versus-host disease
MAT: Medication-assisted treatment
